# High-Intensity Statin Therapy and Associated Rhabdomyolysis in Chronic Liver Disease: A Case Report and Review of Literature

**DOI:** 10.7759/cureus.39150

**Published:** 2023-05-17

**Authors:** Mohan Chandra Vinay Bharadwaj Gudiwada, Vinuthna Gaddam, Maheen Rahman, Jaswanth R Jasti, Paritaben Bhalodia, Sahas Reddy Jitta

**Affiliations:** 1 Internal Medicine, Advocate Illinois Masonic Medical Center, Chicago, USA; 2 Internal Medicine, University of South Dakota Sanford School of Medicine, Sioux Falls, USA; 3 Internal Medicine, Osmania Medical College, Hyderabad, IND

**Keywords:** statin intolerant patients, high-statin therapy, chronic liver disease (cld), statin induced rhabdomylosis, : acute kidney injury

## Abstract

Current literature suggests an increased incidence of rhabdomyolysis in patients with chronic liver disease (CLD) compared to the general population. We present a case of a 60-year-old female with a history of non-alcoholic fatty liver disease and cirrhosis who developed rhabdomyolysis and acute kidney injury after starting high-intensity atorvastatin therapy. This case highlights the potential risks associated with high-intensity statin therapy in patients with CLD, particularly those with advanced liver dysfunction, emphasizing the need for cautious prescribing and thorough risk-benefit assessment in this vulnerable patient population.

## Introduction

Statins are the most prescribed lipid-lowering drugs in the world that have proven benefits in decreasing atherosclerotic cardiovascular diseases. High-intensity statin therapy is associated with a 55-60% reduction in LDL levels and a decrease in major adverse cardiovascular events [1]. Independent of reduction in LDL, statin may also be beneficial in various other diseases including chronic liver disease (CLD) due to its pleiotropic effects [2,3]. Though rhabdomyolysis is a known side effect of statin, its incidence is very low (about 0.1%) [1]. Given that most of these studies excluded individuals with liver disease in their studies, they cannot be generalized to individuals with CLD. There is literature that suggests an increased risk of muscle injury in this subset of patients [4-6]. With the emergence of this new literature, it is important for clinicians to look at its safety in this subset of patients.

## Case presentation

A 60-yrs-old female with a body mass index of 32.77 kg/m^2^, a history of non-alcohol fatty liver disease and cirrhosis (Child-Pugh C), paroxysmal atrial fibrillation, moderate mitral and aortic stenosis, and moderate aortic regurgitation presented to hospital with severe weakness in the bilateral upper and lower extremities especially proximal muscles and an episode of recent mechanical fall. Physical exam revealed ascites and follow-up labs were suggestive of acute kidney injury (AKI), with elevated bilirubin, AST, and mildly elevated ALT. Creatinine phosphokinase (CPK) was found to be 35,400 units/L. An initial diagnosis of rhabdomyolysis was established. Elevated AST was thought to be due to muscle injury. The initial set of labs and home medication are shown in the table below (Table 1).

**Table 1:** Labs on admission and medications prior to admission MELD-NA: 28 Child-Pugh score: 10 points, Class C BUN: blood urea nitrogen, Cr: creatinine, GFR: glomerular filtration rate, AST: aspartate aminotransferase, ALT: alanine transaminase, ALP: alkaline phosphatase, A/G: albumin/globulin, ESR: erythrocyte sedimentation rate, CRP: C-reactive protein, CPK: creatine phosphokinase, MCV: mean corpuscular volume, MCH: mean corpuscular hemoglobin, MCHC: mean corpuscular hemoglobin concentration, RDW: red blood cell distribution width, NRBC: nucleated red blood cells, PT: prothrombin time, PTT: partial thromboplastin time, INR: international normalized ratio, OD: once daily, TID: three times a day, BID: twice a day

METABOLIC PANEL		COMPLETE BLOOD PICTURE		HOME MEDICATION
Sodium	138 mmol/L	WBC	11.4 K/mcL	Aspirin 81 mg OD
Potassium	3.8 mmol/L	RBC	3.60 mil/mcL	Atorvastatin 80 mg OD
Chloride	103 mmol/L	Hemoglobin	12.2 g/dl	Chlorthalidone 25 mg OD
CO2	24 mmol/L	Hematocrit	35.4 %	Famotidine 20 mg OD
Anion Gap	15 mmol/L	MCV	98.3 fl	Insulin glargine 10 Units OD
Glucose	215 mg/dl	MCH	33.9 pg	Lactulose 5g TID
BUN	54 mg/dl	MCHC	34.5 g/dl	Metoprolol succinate 50 mg OD
Creatinine	2.46 mg/dl	RDW-SD	50.7 fl	Rifaximin 550 mg BID
BUN/Cr	22	RDW-CV	14.2 %	Warfarin Sodium 2 mg OD
GFR	21 mL/min/1.73 m2	Platelet	154 K/mcL	
Calcium	8.7 mmol/L	NRBC	0	
Magnesium	2.9 mmol/L	Neutrophil	64 %	
Total Bilirubin	2.3 mg/dL	Lymphocytes	26 %	
Direct Bilirubin	0.5 mg/dL	Monocytes	9 %	
AST	1211 Units/L	Eosinophil	0 %	
ALT	383 Units/L	Basophil	1 %	
ALP	210 Units/L	COAGULATION PANEL		
Globin	4.2 g/dL	PT	23.1 sec	
A/G Ratio	0.4	INR	2.2	
Total Protien	7.4 g/dL	PTT	37 sec	
OTHER LABS				
ESR	2 mm/hr			
CRP	4.7 mg/dl			
CPK	35400 Units/L			

Urinalysis showed muddy casts with high fractional excretion of sodium likely suggestive of intrinsic renal injury. The hepatorenal syndrome was considered differential but muddy casts in UA, current rhabdomyolysis, and lack of response to 2g/kg albumin infusion for two days were suggestive of acute tubular necrosis due to rhabdomyolysis. 25 days prior to hospital admission, she was placed on high-intensity statin therapy (atorvastatin 80 mg) for microvascular ischemic changes seen on the CT head. Atorvastatin was discontinued on the second day of admission. Resuscitative IV fluids, dextrose, and bicarbonate drips were initiated and titrated to alkalinize the urine. CPK was initially up-trending but then started trending down after four days of continuous IV fluids and cessation of atorvastatin. Given the severity of the presentation and proximal weakness, she was also tested for autoimmune myopathies and statin-induced necrotizing rhabdomyolysis, the workup for which has been negative (Table 2).

**Table 2:** Autoimmune/myositis testing panel

ANTINUCLEAR ANTIBODY SCREEN WITH ANTIBODY AND IFA REFLEX	Negative
ANTI SMOOTH MUSCLE ANTIBODY, F ACTIN:	Negative
ANTI-U1-RNP AB	Negative
EJ (GLYCYL-tRNA SYNTHETASE) ANTIBODY	Negative
FIBRILLARIN (U3/RNP)	Negative
HMG-CoA Reductase Ab, S	Negative
JO 1 (Histidyl- tRNA Synthetase)	Negative
JO 1 ANTIBODY IgG	Negative
KU ANTIBODY	Negative
MDA5 (CADM-140) ANTIBODY	Negative
Mi-2 (NUCLEAR HELICASE PROTEIN)	Negative
Mitochondrial ANTIBODY	Negative
NXP2 (NUCLEAR MATRIX PROTEIN-2) (P140) ANTIBODY	Negative
OJ (ISOLEUCYL-tRNA SYNTHETASE) ANTIBODY	Negative
P155/140 ANTIBODY	Negative
PL-12 (ALANYL-tRNA SYNTHETASE) ANTIBODY	Negative
PL-7 (THREONYL-tRNA SYNTHETASE) ANTIBODY	Negative
PM/SCL 100 ANTIBODY, IGG	Negative
RNP ANTIBODY	Negative
SAE1 (SUMO ACTIVATING ENZYME) ANTIBODY:	Negative
SM ANTIBODY	<0.2 AI
SM/RNP ANTIBODY IGG	<0.2 AI
Smith/RNP (ENA) ANTIBODY, IgG	6 Units
SRP (SIGNAL RECOGNITION PARTICLE) ANTIBODY	Negative
SSA-52 (RO52) (ENA) ANTIBODY, IGG	0 AU/mL
SSA-60 (RO60) (ENA) ANTIBODY, IGG	0 AU/mL
TIF-1 GAMMA (155 KDA) ANTIBODY	Negative
U2 SNRNP	Negative

**Figure 1:**
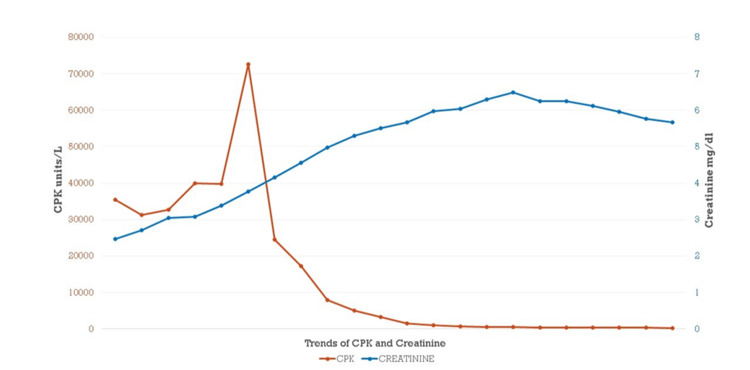
Trend of CPK and creatinine

Over the course of the next few days, her CPK continued to trend down, and LFTs remained stable, but her renal function continued to worsen (Figure 1). After shared decision-making, considering her prior co-morbidities including liver cirrhosis, the patient and family decided not to pursue dialysis. She was managed conservatively with albumin infusions and diuretics. Over the next few days, she developed type II myocardial infarction due to volume overload and disseminated intravascular coagulation. With the progressive decline, the family elected to pursue palliative care.

## Discussion

Statins are well-studied lipid-lowering agents, and the incidence of serious muscle injuries along with rhabdomyolysis is reported as 0.1% [1]. Statin-associated rhabdomyolysis was first reported in 1988 in a cardiac transplant recipient taking lovastatin [7]. Since then, there were many studies on the propensity of these drugs that cause rhabdomyolysis, but most of these did not include patients with liver disease. Though the exact mechanism remains unknown, statins are believed to induce skeletal muscle necrosis likely secondary to a decrease in ubiquinone (coenzyme Q) [8]. Plasma levels, type (hydrophilic vs hydrophobic), dose and drug interactions, and pharmacokinetics [9,10] of statins can each affect the adverse events contributed by the medication. Statins like simvastatin (dose restricted to 40 mg), rosuvastatin (dose restricted to 80mg), pitavastatin (dose restricted to 4mg), and cerivastatin (recalled from the market) were either dose restricted or recalled from the market secondary to increased muscle-related adverse events [1].

Though the incidence of serious muscle injury is minimal in the general population, the incidence tremendously increases in individuals with CLD [4]. In LiverHope, a safety double-blind randomized clinical trial evaluated the safety of different doses of simvastatin along with rifampicin in patients with decompensated cirrhosis and found that 3 of 16 patients (19%) using simvastatin 40 mg + rifampicin 1200 mg developed rhabdomyolysis [5]. The BLEPS (bleeding prevention with simvastatin) trial evaluated the addition of simvastatin to standard therapy in patients with cirrhosis for preventing variceal rebleeding in which 2 of 69 patients (2.9%) on simvastatin developed rhabdomyolysis [6]. In a prospective uncontrolled study evaluating the safety of chronic simvastatin treatment in decompensated liver cirrhosis, 4 of 30 patients (13.3%) developed rhabdomyolysis [4]. This increased incidence of rhabdomyolysis compared to the general population is likely due to increased therapeutic concentration of statin secondary to altered pharmacokinetics in patients with liver disease [4]. Atorvastatin undergoes extensive first-pass metabolism with a bioavailability of around 14% and is dependent on hepatic clearance [11]. Though there are no studies looking at the pharmacokinetics of statin in Child-Pugh Class C patients, it is likely that the bioavailability is much higher in these patients owing to impaired pharmacokinetics.

A meta-analysis done by Kamal et al. showed that the use of statin in liver disease has mortality benefits [3], but most of the studies included in this meta-analysis are observational studies and patients belong to Child-Pugh Class A. This cannot be generalized to all patients with CLD and especially individuals with Child-Pugh Class C. In summary, patients with CLD may be at higher risk of serious muscle injury compared to the general population. Our patient had a CLD with Child-Pugh Class C. The use of high-intensity atorvastatin has led to rhabdomyolysis and AKI.

## Conclusions

The risk versus benefits of statin therapy needs to be thoroughly discussed in patients with liver disease. In patients with CLD, it is advisable to start with a low-intensity statin rather than a high-intensity statin, given that potential side effects are related to the plasma concentration of the drug. In addition, renally metabolized statins can be considered over liver-metabolized statins like atorvastatin. Further research is needed to establish the true incidence of rhabdomyolysis and the safety of atorvastatin in patients with CLD.
